# Saikosaponin-d Attenuates Hashimoto's Thyroiditis by Regulating Macrophage Polarization

**DOI:** 10.1155/2022/7455494

**Published:** 2022-11-08

**Authors:** Peng Du, Jianbin Xu, Yanfei Jiang, Jing Zhao, Chaoqun Gao, Yudie Fang, Xiaorong Yang, Yan-ping Yang, Jin-an Zhang

**Affiliations:** ^1^Department of Endocrinology & Rheumatology, Shanghai University of Medicine & Health Sciences Affiliated Zhoupu Hospital, Shanghai 201318, China; ^2^Graduate School, Shanghai University of Traditional Chinese Medicine, Shanghai 201203, China; ^3^Shanghai University of Traditional Chinese Medicine, Shanghai 201203, China

## Abstract

**Objective:**

Hashimoto's thyroiditis (HT) is one of the most common clinical autoimmune diseases. Recent studies have found that HT pathogenesis is associated with macrophage polarization. Saikosaponin-d (SSd) is an active component in the Chinese medicine Bupleurum, which has anti-inflammatory and immunomodulatory effects. The purpose of this study was to verify the therapeutic effect of SSd on HT and to investigate the regulatory effect of SSd on macrophage polarization in HT.

**Methods:**

Network pharmacology analysis was used to predict the relevant targets and signaling pathways of SSd for HT treatment. The therapeutic effect of SSd on HT model mice and the effect on macrophage polarization were detected by animal experiment.

**Results:**

Network pharmacological analysis showed that SSd can alleviate HT against multiple targets such as IL-6 and IL-10 and can act on macrophage polarization-related signaling pathways such as MAPK and JAK-STAT signaling pathways. Animal experiments showed that SSd intervention attenuated the lymphocytic infiltration in thyroid tissues of HT mice (*P* = 0.044); SSd intervention reduced serum TPOAb antibody level in HT mice (*P* < 0.001); SSd adjusted M1/M2 imbalance towards M2-type macrophage polarization in the spleen of HT mice (*P* = 0.003); SSd inhibited the expressions of Th1-type cytokine IFN-*γ* and Th17-type cytokine IL-17 systemically and locally in the thyroid of HT mice (*P* < 0.05).

**Conclusion:**

SSd treatment can regulate Th1/Th2 and Th17/Treg imbalances and reduce the severity of HT in mice by promoting the polarization of M2 macrophages.

## 1. Introduction

Hashimoto's thyroiditis (HT), also known as chronic lymphoid thyroiditis, lymphoid goiter, or autoimmune thyroiditis, is one of the most common clinical autoimmune diseases, characterized by thyroid enlargement, lymphocyte infiltration, and the occurrence of thyroid antigen-specific antibodies. HT is currently the main cause of hypothyroidism [1, 2]. What is more, patients with HT are more likely to suffer from cardiovascular disease and malignancy [3, 4]. In recent years, the incidence of HT has increased significantly. Epidemiological studies show that the prevalence of HT in China is over 10%, and the total number of patients is over 130 million [5]. HT is generally considered to be a common organ-specific autoimmune disease due to the interplay of genetic and environmental factors. Its main immune abnormalities are B cell dysfunction and production of thyroid autoantibodies, and abnormal T cell subsets also play a key role in breaking immune homeostasis and causing damage to thyroid tissues [6–9]. HT patients generally express high levels of anti-thyroglobulin antibodies (TgAb) and thyroid peroxidase antibody (TPOAb) in serum, which are produced by B cells after stimulation with the immunogenicity of thyroglobulin and thyroid peroxidase and mediated by T lymphocytes.

In addition, T cells can not only promote B cells to release autoantibodies but also release proinflammatory cytokines and maintain the number of autoreactive memory T cells, thus enhancing the autoimmunity against thyroid tissues [6–8, 10]. In HT, CD4+ helper T (Th) cells play a key role in the occurrence of disease. The balance between effector T cells and regulatory T (Treg) cells is the key to the immune tolerance of thyroid tissues, and its imbalance can lead to the development of HT [9, 11, 12]. The effects of Th1 and Th2 on HT have been recognized in previous studies [13–16]. Tregs exert immunosuppressive effects by secreting TGF-*β* and IL-10. Th17 cells, with signal transduction transcription factor and activator of transcription 3 (STAT3) and retinoic acid-associated orphan receptor *γ*t (ROR*γ*t) as transcription factors, can secrete interleukin- (IL-) 17 and mediate inflammatory response [17].

Besides T cells and B cells, macrophages, as an antigen-presenting cell, are an important part of the human immune response. Recent studies have shown that macrophages can undergo phenotypic and functional changes in two different directions under environmental induction, namely, M1 macrophages (IFN-*γ* and LPS-induced proinflammatory effect) and M2 macrophages (IL-4- and IL-13-induced anti-inflammatory and tissue repair effects). This process is known as macrophage polarization. The imbalance of macrophage polarization is involved in the pathogenesis of various autoimmune diseases, including HT [18, 19].

Saikosaponin-d (SSd) is an important and effective compound extracted from the root of Bupleurum and is the most active and widely studied triterpenoid saponin in Chinese medicine Bupleurum [20]. SSd has anti-inflammatory, immune regulation, anticancer, and other functions. Among them, the anti-inflammatory and immunomodulatory functions of SSd are widely used and have been studied in a variety of autoimmune diseases [21, 22]. In addition, studies have shown that SSd inhibits M1 macrophage polarization and proinflammatory cytokine production via the nuclear factor-*κ*B (NF-*κ*B) pathway [23]. The aim of this study was to determine the therapeutic effect of SSd on HT and the effect of SSd on macrophage polarization and related cytokines in an animal model of HT.

## 2. Materials and Methods

### 2.1. Network Pharmacology Analysis

HT-related genes were obtained from the NCBI Gene database. 2D structure and 3D structure files corresponding to SSd were obtained from PubChem database, as well as the action targets published in literature. SwissTargetPrediction database and SEA (similarity analysis of chemical structure) database were used to predict drug targets, and PharmMapper database was used to predict pharmacology-based drug action targets. The above targets were screened and summarized. HT-related genes and SSd targets were transformed into UniProt ID using UniProt database, and then, the two types of targets were compared and analyzed. STRING database and Cytoscape 3.6.0 software were used to analyze the network structure and screen hub genes. HiPlot tool was used to conduct KEGG signal pathway and GO biological process (BP) enrichment analysis for screened hub genes. KEGG pathway enrichment analysis was used to analyze the related signal pathway of SSd action on HT, and GO BP enrichment analysis was used to analyze the related biological process of SSd action on HT, so as to clarify the effect of SSd on HT.

### 2.2. Animal Model

The ethics committee of Zhoupu Hospital affiliated to Shanghai Health Medical College approved the animal experiment (No. 2018-C-027-E01). SSd was purchased from Shanghai Tauto Biotech Co., Ltd. 36 female wild-type C57BL/6N mice aged 6-8 weeks were purchased from Zhejiang Vital River Laboratory Animal Technology Co., Ltd. and randomly divided into three groups according to body weight using SPSS software: normal control (NC) group (*n* = 10), HT modeling (HT) group (*n* = 10), and SSd intervention (SSd) group (*n* = 16). HT and SSd groups were injected with pig thyroglobulin (100 *μ*g/animal) once a week, and thyroglobulin for injection in the first week was emulsified with complete Fredrin's adjuvant, and thyroglobulin for injection in the remaining 5 weeks was emulsified with incomplete Fredrin's adjuvant and was injected subcutaneously in the neck, abdomen, and back (NC group was injected with normal saline at the same site). The HT group and SSd group were fed 0.05% NaI high iodine water throughout the whole process, and the NC group was fed normal water and feed, without drug intervention. One week before the induction of HT disease model, the HT group was given 0.2 mL of 0.5% sodium carboxymethyl cellulose (CMC) solution every other day, and the SSd group was given SSd (5 mg/kg) dissolved by 0.5% sodium CMC solution every other day. After 6 weeks of animal modeling, mice were sacrificed with isoflurane, and their serum, spleen, and thyroid tissues were collected.

### 2.3. Antibody and TSH Detection

Peripheral blood of mice was collected into 1.5 mL tubes containing heparin sodium. The blood samples were centrifuged at 450 g for 5 min at 4°C; then, the supernatant was collected and stored at -80°C for antibody and TSH determination. TPOAb, TgAb, and TSH were detected by ELISA using mouse TPOAb, TgAb, and TSH ELISA kits (Elabscience, Wuhan, China).

### 2.4. Thyroid Histopathology

The thyroid tissue was fixed in 4% paraformaldehyde and made into 4 *μ*m paraffin sections. The sections were stained with hematoxylin-eosin (HE), observed under a microscope, and scanned with a scanning system to preserve the whole section.

Three discontinuous areas were randomly selected in each section, and the degrees of thyroid lymphocyte infiltration were scored and averaged. The scoring criteria were as follows: no lymphocytic infiltration in the visual field was 0; the area of lymphocyte infiltration in the visual field was less than 10%, which was 1 point. The area of lymphocyte infiltration in the field of vision was 2 points in 10%~30%. The area of lymphocyte infiltration in the field of vision was 3 points in 30-50%. The area of lymphocyte infiltration above 50% in the visual field was 4 points.

### 2.5. Flow Cytometry

Mouse spleen cells were obtained by grinding mouse spleens, part of which was prepared into suspension of 10^6^ cells/mL. The cells were subjected to antibody staining with PE-Cy7-anti-CD11B, AF-647-anti-F4/80, fluorescein isothiocyanate- (FITC-) anti-CD86, and phycoerythrin- (PE-) anti-CD206, in which PE-Cy7-anti-CD11b and AF-647-anti-F4/80 antibodies were used to detect macrophages, FITC-anti-CD86, and PE-anti-CD206 differentiated M1 and M2 phenotypes. Finally, CD11b+F4/80+CD86+CD206- represents M1 macrophages and CD11b+F4/80+CD86-CD206+ represents M2 macrophages.

### 2.6. Real-Time Quantitative Polymerase Chain Reaction

Part of the spleen cells was lysed with TRIzol reagent (Takara, Dalian, China) and stored at -80°C for total RNA extraction. cDNA was synthesized by reverse transcription (RT) using the PrimeScript RT kit (Takara, Dalian, China). The relative expression levels of Tnf, Il1b, Il6, Il10, Il17, Ifng, Tbet, and Gata3 genes in mice spleen cells were detected by real-time quantitative polymerase chain reaction (RT-qPCR). RT-qPCR was performed using the ABI 7500 real-time quantitative PCR system (Applied Biosystems, Inc., Foster City, California) under the following conditions: 95°C reaction 30 seconds, 95°C reaction 5 s, and 63°C reaction 34 s, 45 times, and then, the reaction at 95°C for 15 s, 60°C for 1 min, 95°C for 15 s, and 60°C for 15 s, once. GAPDH was an internal reference gene. The mRNA relative expression levels of target genes were calculated and detected by the 2^-*ΔΔ*CT^ method.  Table 1 shows the primer sequences.

### 2.7. Immunohistochemical Staining

Part of mouse thyroid tissue was prepared into paraffin sections (4 *μ*m) and immunohistochemical staining was performed using classical methods. The rat anti-mouse IFN-*γ* antibody (Servicebio, Wuhan, China) was used at 1 : 100 dilution, and the second antibody was IgG conjugated with horseradish peroxidase-labeled streptavidin in goat-resistant rats (Servicebio, Wuhan, China). After staining, the sections were scanned and the IFN-*γ* positive area was calculated using ImageJ software.

### 2.8. Data Analysis

All data were analyzed by SPSS 23.0 and GraphPad Prism 8.0 software was used to complete the mapping. Continuous variables were expressed by mean ± standard deviation (mean ± SD). The data were tested for normality before comparing two groups of continuous variables, and the data for both groups of continuous variables were normally distributed using the *t*-test, and if one group or more of data did not conform to the normal distribution, the Mann-Whitney *U* test was used. *P* < 0.05 indicates a statistical difference.

## 3. Results

### 3.1. Network Pharmacology Analysis of Saikosaponin-d on HT

**Figure 1:**
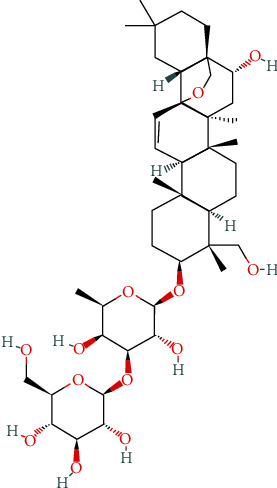
SSd 2D chemical structure.

One hundred and nine HT targets were screened through NCBI Gene database, as shown in Table 2. The simplified molecular input line entry system (SMILES) of SSd was obtained using PubChem database: CC1C(C(C(C(O1)OC2CCC3(C(C2(C)CO)CCC4(C3C=CC56C4(CC(C7(C5CC(CC7)(C)C)CO6)O)C)C)C)O)OC8C(C(C(C(O8)CO)O)O)O)O. Download the 2D SSd structure (Structure2D_CID_107793), as shown in Figure 1. 100 SSd-related genes found in the PubChem database were queried, and 100 SSd-related genes were predicted by inputting SMILES using the SwissTargetPrediction, including 16 genes with probability > 0.5; SSd-related genes were predicted by SMILES input in SEA database. Using the PharmMapper server database to upload the SSd 2D structure file, 299 SSd-related genes were predicted and 129 genes with norm fit > 0.8 were screened. The 4 database records and predicted genes were summarized, and a total of 126 unique SSd-related gene targets were obtained, as shown in Table 2.

**Figure 2:**
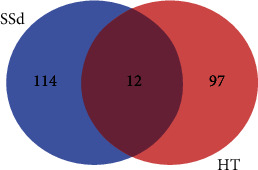
Number of HT and SSd-related gene targets.

**Figure 3:**
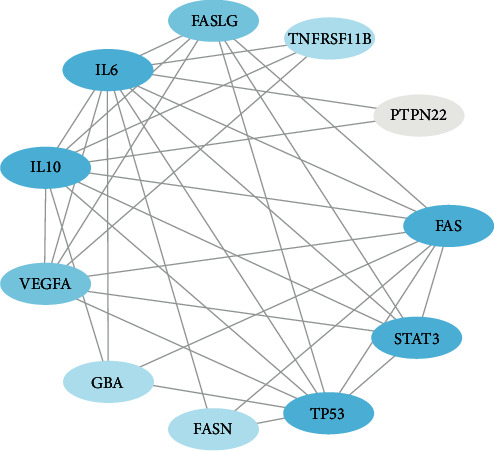
Protein-protein interactions of SSd-HT targets.

12 targets were obtained after the intersection of SSd-predicted targets and HT-related targets, as shown in Tables 2 and 3 and Figure 2. We imported the targets into the STRING database, used the database to construct the relevant protein-protein interaction network, selected *Homo sapiens* as the species, and obtained the protein interaction network. Then, we downloaded the TSV data file that saves the interaction network and imported it into Cytoscape 3.6.0 software to get the network-related information. The number of nodes in this network is 11, the number of edges is 31, the average degree of nodes is 6, and the average local clustering coefficient is 0.848. There is no direct interaction between GC and other target proteins, so it is not shown in the network topology analysis. We also calculated the following values for each node using the NetworkAnalyzer tool in Cytoscape (Table 4): the average shortest path length (ASPL), the closeness centrality (CC), the clustering coefficient, and the degree. ASPL represents the minimum number of links a node needs to connect to the entire network. In a network, the smaller the ASPL, the more efficient the signal transduction through nodes. CC and clustering coefficient can reflect the tightness of the network, and the tighter the network, the higher the efficiency [24, 25]. The degree of nodes refers to the number of edges between nodes in the network, usually indicating the importance of each node in the network [26, 27]. The degree value is represented by color depth, and all nodes are sorted according to degree value. The closer the color is to blue, the greater degree value is, as shown in Figure 3.

**Figure 4:**
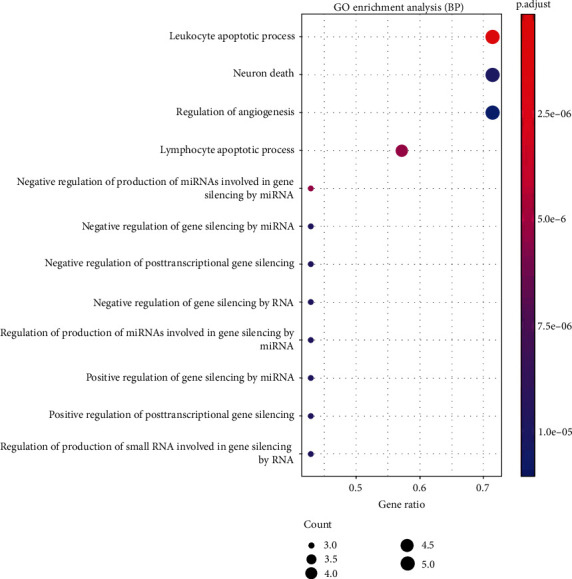
GO enrichment analysis of the SSd-HT hub genes.

**Figure 5:**
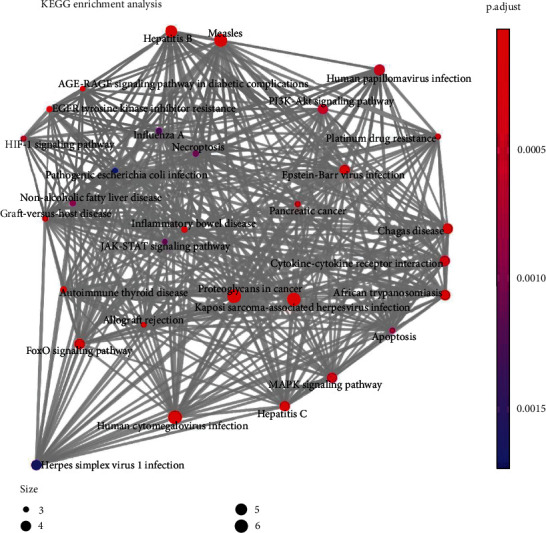
KEGG enrichment analysis of the SSd-HT hub gene.

To further clarify the mechanism of SSd improving HT, targets with degree value ≥ 6 were selected as hub genes for enrichment analysis of GO and KEGG pathways, and bubble and mesh diagrams were drawn, as shown in Figures 4 and 5. GO enrichment analysis showed that SSd was involved in leukocyte apoptosis, neuronal death, angiogenesis regulation, lymphocyte apoptosis, negative regulation of miRNA, production of miRNA involved in gene silencing, and other inflammatory and immunobiological processes. KEGG pathway enrichment analysis showed that SSd mechanism involved MAPK signaling pathway, JAK-STAT signaling pathway, PI3K-Akt signaling pathway, and other common signaling pathways as well as inflammatory bowel disease, diabetes, autoimmune thyroid disease, and other related pathways.

### 3.2. Evaluation of Disease in HT Model Mice and Saikosaponin-d-Treated Mice

**Figure 6:**
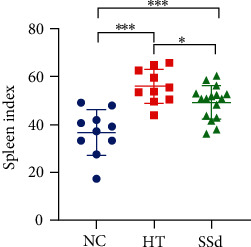
Mice spleen index (^∗∗∗^*P* < 0.001, ^∗^*P* < 0.05).

**Figure 7:**
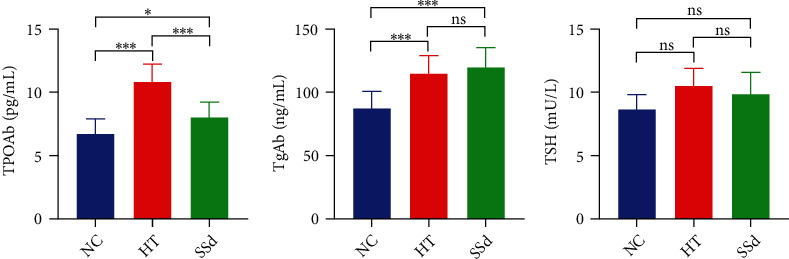
Serum TPOAb, TgAb, and TSH concentrations in mice (^∗∗∗^*P* < 0.001).

**Figure 8:**
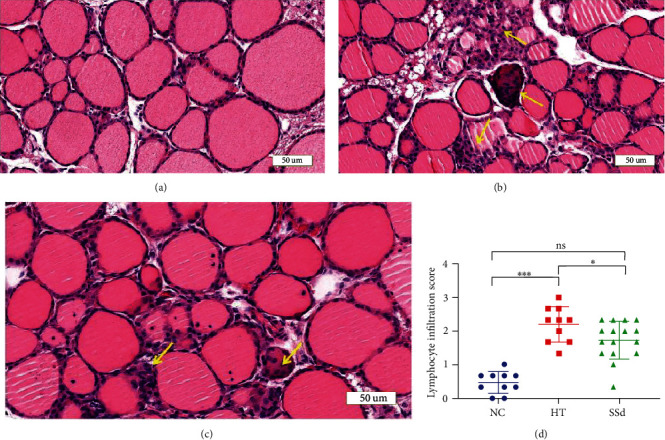
Thyroid lymphocyte infiltration in mice. (a) HE staining of thyroid tissue in the NC group mice (200x). (b) HE staining of thyroid tissue in the HT group mice (200x). (c) HE staining of thyroid tissue in the SSd group mice (200x). (d) Comparison of thyroid lymphocyte infiltration scores in mice (^∗∗∗^*P* < 0.001, ^∗^*P* < 0.05). Lymphocytic infiltration is evident where the yellow arrow points.

In this study, the disease severity of HT model mice and SSd-treated mice was mainly assessed from the spleen index, thyroid lymphocyte infiltration degree, and serum TPOAb, TgAb, and TSH concentrations. The spleen index of the HT group was higher than that of the NC group (*P* < 0.001); the spleen index of the SSd group was lower than that of the HT group (*P* = 0.028), as shown in Figure 6. ELISA results showed that the serum TPOAb concentration of HT mice was higher than that of the NC group (*P* < 0.001), while SSd intervention reduced serum TPOAb antibody level in mice (*P* < 0.001); compared with the NC group, the serum TgAb concentration of HT mice was higher (*P* < 0.001), while there was no significant difference between the SSd group and HT group. There was no significant difference between the NC group, HT group, and SSd group in serum TSH levels, as shown in Table 5 and Figure 7. As shown in Figure 8, HE staining revealed significant lymphocyte infiltration and changes in follicular morphology in thyroid tissues under pathological conditions of the HT modeling group. There was significant difference in lymphocyte infiltration score between the HT group and the NC group (*P* < 0.001), and the infiltration degree of thyroid lymphocytes in SSd group was significantly reduced compared with the HT group (*P* = 0.044). All data conformed to a normal distribution and were analyzed by *t*-test.

### 3.3. Cell and Cytokine Levels of HT Model Mice and Saikosaponin-d-Treated Mice

**Figure 9:**
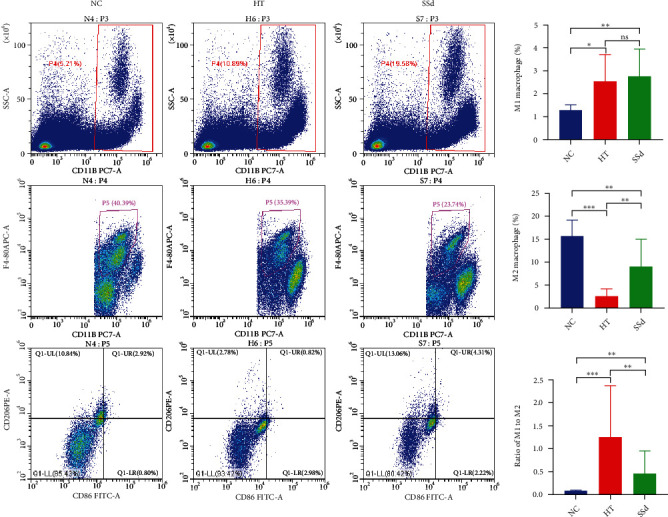
Flow cytometric detection of M1/M2 macrophages in mice spleen (^∗∗∗^*P* < 0.001, ^∗∗^*P* < 0.01, ^∗^*P* < 0.05).

In order to further explore the changes in immune status in HT and the mechanism of SSd intervention in improving HT, we detected the expression levels of spleen immune cells, mRNA, and thyroid tissue protein by flow cytometry, real-time qPCR, and immunohistochemical staining. The results showed that the proportion of M1 macrophages in total macrophages in the HT group was significantly higher than that in the NC group (*P* = 0.016), and there was no significant difference between the SSd intervention group and HT group; the proportion of M2 macrophages in the HT group was significantly lower than that in the normal group (*P* < 0.001), while the proportion of M2 cells in the SSd intervention group was higher than that in the HT group (*P* = 0.003). The M1/M2 ratio in the HT group was significantly higher than that in the NC group (*P* < 0.001), while the M1/M2 ratio was significantly decreased by the SSd intervention (*P* = 0.004), as shown in Figure 9 (Mann-Whitney *U* test and *t*-test were both used).

**Figure 10:**
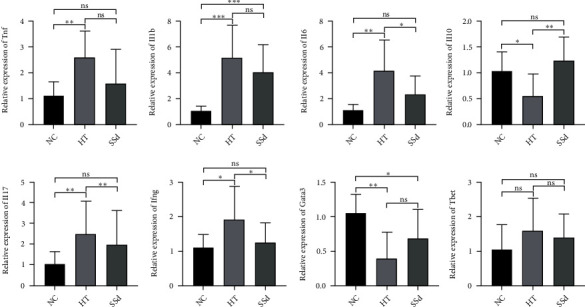
Expression levels of mRNA in mice spleen cells (^∗∗∗^*P* < 0.001, ^∗∗^*P* < 0.01, ^∗^*P* < 0.05).

As shown in Figure 10, RT-qPCR results showed that the mRNA expression levels of Il6, Il17, and Ifng in spleen cells in the HT group were higher than those in the NC group (*P* = 0.003; *P* = 0.003; *P* = 0.024) and SSd intervention reduced the mRNA levels of Il6, Il17, and Ifng (*P* = 0.043; *P* = 0.004; *P* = 0.048); compared with the NC group, the mRNA expression levels of Il1b and Tnf in the HT group were higher (*P* < 0.001; *P* = 0.002), but there was no significant difference between the SSd group and the HT group in Il1b and Tnf mRNA expressions. Compared with the NC group, the mRNA expression level of Il10 in the HT group was decreased (*P* = 0.032), and that in the SSd group was higher than the HT group (*P* = 0.004). Compared with the NC group, Gata3 mRNA expression level in HT group was significantly decreased (*P* = 0.003), but there was no significant difference in Gata3 mRNA expression level between the SSd group and HT group. There was no significant difference in Tbet mRNA expression between the NC group, HT group, and SSd group. The Mann-Whitney *U* test and *t*-test were both used.

**Figure 11:**
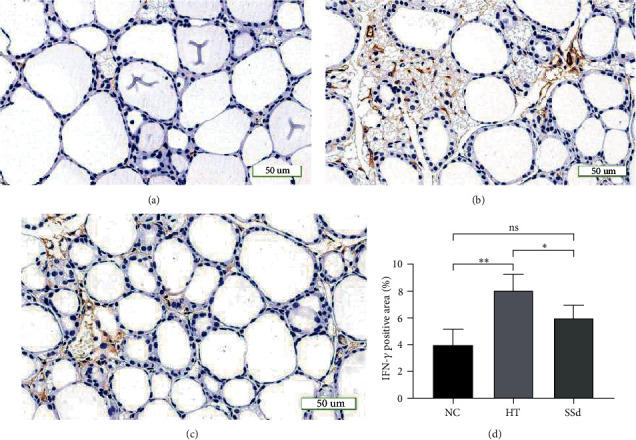
Expression of IFN-*γ* protein in the mice thyroid. (a) Immunohistochemical staining of thyroid tissue in the NC group of mice (200x). (b) Immunohistochemical staining of thyroid tissue in the HT group of mice (200x). (c) Immunohistochemical staining of thyroid tissue in the SSd group of mice (200x). (d) Comparison of the area (%) positive for IFN-*γ* protein in mice thyroid tissue (^∗∗^*P* < 0.01, ^∗^*P* < 0.05).

Immunohistochemical staining was used to detect the expressions of IFN-*γ* protein in the thyroid tissues of mice in the two groups. The results showed that the positive area of IFN-*γ* protein in the thyroid tissues of mice in the HT group was significantly increased compared with that in the NC group (*P* = 0.003). Compared with the HT group, the positive expression of IFN-*γ* in the thyroid tissues of mice in the SSd intervention group decreased (*P* = 0.042) (as shown in Figure 11). All data conformed to a normal distribution and were analyzed by *t*-test.

## 4. Discussion

HT, as an inherited autoimmune disease, is characterized by the destruction of thyroid cells by cellular and antibody-mediated immune responses [28]. HT is the most common cause of hypothyroidism in developed countries [29]. However, effective treatment methods for HT are very limited. Control of hypothyroidism is the main method and purpose of treatment, and the main drug therapy is oral synthetic hormone levothyroxine [30]. Currently, there is a lack of specific treatments that block the immune response and prevent the disease from progressing to hypothyroidism. Network pharmacology is an emerging strategy to study the interaction between drugs and diseases systematically and holistically, which can clarify the synergistic effects of various compounds and their potential mechanisms by analyzing multilevel interaction networks [31]. At present, network pharmacology has been widely applied in the study of traditional Chinese medicine and related mechanisms.

In this study, 12 proteins were obtained from the intersection of SSd prediction targets and HT disease targets, and through the protein-protein interaction network analysis, 7 hub genes including IL-6, IL-10, FAS, TP53, VEGFA, STAT3, and FASLG were selected. Studies have shown that these 7 central hub genes are involved in the pathological process of autoimmune thyroid disease. IL-6 is a typical proinflammatory cytokine that can stimulate T cell proliferation and cytotoxic T lymphocyte activation and is highly expressed in HT [32]. IL-10 is a Th2 cell-associated cytokine playing a protective role in the development of HT [33]. Fas expression exists in thyroid follicular epithelial cells, and Fas/FasL pathway-mediated apoptosis plays an essential role in the development of HT. P53, encoded by the tumor suppressor gene TP53, plays a key role in the mechanisms of cell cycle arrest, apoptosis, and maintenance of genetic integrity in response to a variety of injuries including DNA damage, hypoxia, and metabolic stress. TP53 is not only involved in the pathological mechanism of tumors but also in the development of HT [34]. STAT3 is a transcription factor of Th17 and Tfh cells and regulates their gene expression, while Th17 and Tfh cells both participate in the process of HT disease [12, 35, 36]. VEGFA is a vascular endothelial cell-specific mitogen that can promote the growth of vascular endothelial cells and induce vascular hyperplasia and is widely involved in inflammation [37]. Further, GO enrichment analysis of the above 7 central target proteins suggested that SSd is involved in MAPK, JAK-STAT, PI3K-Akt, and other pathways and plays a role in inflammatory enteritis, diabetes, autoimmune thyroid disease, and other autoimmune diseases. Current studies have shown that JAK-STAT signaling pathway is an important pathway in the polarization of M1 macrophages [38]. The PI3K-Akt signaling pathway participates in the activation and gene expression of macrophages, and the change of signaling factors (such as NF-*κ*B) in the pathway can affect the polarization of macrophages [39]. MAPK signaling pathway is a key participant in the polarization of M2 macrophages [40]. Therefore, SSd may affect macrophage polarization through multiple signaling pathways, thus achieving the effect of alleviating HT.

In this study, we determined whether HT was successfully modeled by the primary indicators of whether TPOAb and TgAb were elevated in serum and lymphocyte infiltration in thyroid tissue, and the secondary indicator was TSH level. This is consistent with the clinical diagnostic criteria for patients with HT. This is because during the progression of HT, patients initially present with elevated thyroid-related antibodies and diffuse enlargement of the thyroid gland, which may be without changes in thyroid function. In later stages of disease progression, hypothyroidism may occur, i.e., elevation of TSH and decrease in FT3 and FT4, with TSH often preceding FT3 and FT4. In the present study, due to the short modeling time, although hypothyroidism did not occur in the modeled mice (although TSH levels tended to increase), there were significant changes in the main indicators, and these results indicate that our modeling was successful. Furthermore, the aim of this study was the potential immunomodulatory effect of the herbal monomer on HT, rather than the improvement of thyroid function. This is also consistent with clinical experience with medication (currently, only L-T4 improves hypothyroidism).

Actually, with the means of flow cytometry, our results showed that there were significantly lower proportion of M2 macrophages and higher proportion of M1 macrophages in the spleen of HT mice. SSd intervention enhanced M2 macrophage proportion and decreased M1/M2 ratio significantly. There was direct evidence that SSd alleviates the pathological changes of HT. SSd intervention reduced lymphocytic infiltration and INF-*γ* expression in thyroid tissue of HT mice and decreased the concentration of thyroid autoantibodies in serum.

The detection of cytokine expression in spleen cells also proved the immunointerventional effects of SSd on HT. It has long been established that the Th1/Th2 imbalance presents a Th1 dominance in HT scenario. IFN-*γ* is a typical proinflammatory cytokine secreted by Th1 cell, while IL-10, as an important anti-inflammatory cytokine, is significantly associated with Th2 cells, Treg cells, and M2 macrophages. IFN-*γ* can inhibit the cotransport of sodium and iodine in thyroid follicular epithelial cells, upregulate the expression of Fas on cell surface, and lead to cell apoptosis [41]. Some studies have suggested that IFN-*γ* can be used as a reliable marker of HT [42]. Our in vivo experiment showed that IFN-*γ* level in the SSd intervention group was significantly lower than that in the HT group, while IL-10 level was higher than that in the HT group. Th17 is a relatively newly defined type of T lymphocyte and relies on the production and secretion of IL-17 to play a powerful proinflammatory role. Treg cells, a typical immunomodulatory cell, inhibit Th17 immune response by secreting IL-10. Our animal study not only confirmed the high expression of IL-17 and the low expression of IL-10 in spleen cells of HT mice but also demonstrated that SSd can reduce IL-17 and promote the expression of IL-10. In fact, the effects of SSd on promoting the expression of anti-inflammatory cytokines IL-10 and inhibiting the expression of inflammatory cytokines TNF-*α*, IL-1, and IL-6 have been repeatedly reported in other studies [43]. Thus, in HT, SSd plays a certain role in improving Th1/Th2 and Th17/Treg imbalance and regulating M1/M2 polarization.

In conclusion, SSd treatment can regulate Th1/Th2 and Th17/Treg imbalances and reduce the severity of HT in mice by promoting the polarization of M2 macrophages, thus exerting a preventive and therapeutic effect on HT.

## Figures and Tables

**Table 1:** Primer sequences used for RT-qPCR.

Gene		Primer sequence (5′-3′)	Primer size
Tnf	Forward	ATGTCTCAGCCTCTTCTCATTC	22
	Reverse	GCTTGTCACTCGAATTTTGAGA	22
Il1b	Forward	GAAATGCCACCTTTTGACAGTG	22
	Reverse	TGGATGCTCTCATCAGGACAG	21
Il6	Forward	TAGTCCTTCCTACCCCAATTTCC	23
	Reverse	TTGGTCCTTAGCCACTCCTTC	21
Il10	Forward	GCTCTTACTGACTGGCATGAG	21
	Reverse	CGCAGCTCTAGGAGCATGTG	20
Il17	Forward	TTTAACTCCCTTGGCGCAAAA	21
	Reverse	CTTTCCCTCCGCATTGACAC	20
Ifng	Forward	ACAGCAAGGCGAAAAAGGATG	21
	Reverse	TGGTGGACCACTCGGATGA	19
Tbet	Forward	GTTCAACCAGCACCAGACAGAG	22
	Reverse	TGGTCCACCAAGACCACATC	20
Gata3	Forward	GGATGTAAGTCGAGGCCCAAG	21
	Reverse	ATTGCAAAGGTAGTGCCCGGTA	22
Gapdh	Forward	AGGTCGGTGTGAACGGATTTG	21
	Reverse	TGTAGACCATGTAGTTGAGGTCA	23

**Table 2:** Related gene targets of HT and SSd.

Drug/disease	Targets	Number
SSd	GSTP1, MAPK14, KDR, CA2, SPSB1, MTOR, SLC5A1, MAPK8, ICAM2, HSD17B1, BMP2, EGFR, FGF21, FTO, AMY1A, SPSB2, ACE2, PDE4B, MAPK10, ADORA1, SULT2A1, LGALS7, TNNC1, MAPK3, CYP3A7, DHODH, CA12, ITGAL, AMY1B, BCHE, AMY1C, MMP13, PGR, ANG, HCK, METAP1, KIF11, PIM1, CASP7, TSC22D3, NR1H2, HSD11B1, BACE1, CDK2, NUDT9, MAPK1, CTSD, SEC14L2, LTB4R, ADAM8, EPHB4, MAP2K4, SPSB4, SOCS3, TTR, CHEK1, QPCT, AURKA, CYP1A2, PTAFR, PPIA, FAP, ALB, F3, RB1, CFB, CBL, MAPKAPK2, TREM1, PTPRA, HDAC8, FGF2, RAB5A, AKR1C2, GCK, SMAD7, FCAR, STS, CDKN1A, IMPA1, ABCC1, PTPN2, MAOB, CLPP, TGFBR2, PAH, MTAP, CLC, OPRK1, DDX6, CASP3, METAP2, CALM2, NABP2, FKBP1A, BDNF, DCX, MMP3, APOA2, PDGFRB, HSD17B11, HTR2C, RORA, CA1, FOXG1, CTSV, SLC5A2, FGF1, PNP, F2, NABP1, CYP3A4, RBL2, TFEB	114

SSd-HT	PTPN22, GC, FASN, GBA, IL6, FAS, TP53, TNFRSF11B, STAT3, VEGFA, FASLG, IL10	12

HT	TNFSF12, IL12B, TNFSF8, CALCA, CGA, TGFB1, CXCL10, FOXP3, IL23A, IFNL3, CLDN1, TNFSF18, DIO1, MX1, TNFRSF8, CASP8, EDN1, IL18R1, MAGI2, CD40, RET, AMH, RHO, IL4, IL18RAP, LEP, IL1R1, TNFSF4, AATF, SLC26A4, IL37, IL1RL1, CD80, CD1C, ABCC11, IL17A, TBX21, MBL2, S100A9, DPP4, CALCA, NRAS, PTPRC, COL1A1, HGF, FCGRT, TSHR, PTGDR2, TG, TPO, SH2B3, TNFSF13B, PER3, CCL5, SPP1, SELENOS, KRT19, PDE8B, BRAF, DIO2, TLR2, SMARCA2, IL18, IFNL1, EDNRA, TSHB, FCGR2B, ABCC6, VDR, NAAA, IL33, AGER, IFNG, TNFRSF13C, SLTM, CD1A，TNFAIP3, HLA-A, TNFRSF1A, CXCL9, NFE2L2, ARID5B, IFIH1, HLA-DRB4, IL23R, IL1B, TLR4, SGPP1, SLC30A8, CTLA4, GGCX, CYP27B1, MET, TNF, IL22, NCAM1, IL6R	97

**Table 3:** Intersection of SSd and HT targets.

No.	UniProt ID	Gene name	Protein name
1	P04637	TP53	Tumor protein p53
2	P15692	VEGFA	Vascular endothelial growth factor A
3	P05231	IL6	Interleukin 6
4	P22301	IL10	Interleukin 10
5	Q9Y2R2	PTPN22	Protein tyrosine phosphatase nonreceptor type 22
6	P02774	GC	GC vitamin D binding protein
7	P49327	FASN	Fatty acid synthase
8	P04062	GBA	Glucosylceramidase *β*
9	O00300	TNFRSF11B	TNF receptor superfamily member 11b
10	P40763	STAT3	Signal transducer and activator of transcription 3
11	P48023	FASLG	Fas ligand
12	P25445	FAS	Fas cell surface death receptor

**Table 4:** Topological analysis of SSd-HT target network.

Target name	ASPL	Closeness centrality	Clustering coefficient	Degree
IL6	1	1	0.51111111	10
IL10	1.1	0.90909091	0.58333333	9
FAS	1.2	0.83333333	0.71428571	8
TP53	1.2	0.83333333	0.71428571	8
VEGFA	1.3	0.76923077	0.80952381	7
STAT3	1.4	0.71428571	1	6
FASLG	1.4	0.71428571	1	6
GBA	1.6	0.625	1	4
FASN	1.7	0.58823529	1	3
TNFRSF11B	1.7	0.58823529	1	3
PTPN22	1.8	0.55555556	1	2

**Table 5:** Serum TPOAb, TgAb, and TSH concentrations in mice (mean ± SD).

Group	*n*	TPOAb (pg/mL)	TgAb (ng/mL)	TSH (mU/L)
NC	10	6.72 ± 1.18	89.43 ± 14.24	8.72 ± 1.37
HT	10	10.81 ± 1.41^∗∗∗^	116.6 ± 12.84^∗∗∗^	10.62 ± 2.83
SSd	16	7.97 ± 1.26^###^	121.4 ± 17.96	10.01 ± 2.12

Compared with the NC group, ^∗∗∗^*P* < 0.001; compared with the HT group, ^###^*P* < 0.001.

## Data Availability

The authors confirm that the data supporting the findings of this study are available within the article.
